# Supplementary material for: A novel regulation on the developmental checkpoint protein Sda that controls sporulation and biofilm formation in Bacillus subtilis
Source: J Bacteriol. 2025 Feb 11;207(3):e00210-24. doi: 10.1128/jb.00210-24 (PMC11925247; doi:10.1128/jb.00210-24)
Supplement: Figures S1 and S2 — Fig. S1: Synonymous substitution in serine codons in sda did not impact growth. Fig. S2: Transcription of sda in the wild-type and synonymous serine mutants showed an insignificant difference. [file jb.00210-24-s0001.pdf]

Supplementary figure legends.

Figure S1. Synonymous substitution in serine codons in *sda* did not impact growth.

Growth curve of wild-type and serine mutants in DSM and MSgg medium. Cells of each strain were freshly grown to mid-log phase. For growth in DSM medium, cells were inoculated at a 1:1000 ratio into 100 µL of fresh DSM medium in a 96-well plate. The plate was incubated at 37°C for 15 hours with shaking at 200 rpm in the plate reader. For growth in MSgg, cells were inoculated at a 1:1000 ratio into 20 mL of fresh MSgg medium in a flask. The flasks were incubated at 37°C for 10 hours with shaking at 200 rpm in the incubator.

Figure S2. Transcription of *sda* in the wild-type and synonymous serine mutants showed an insignificant difference.

Fresh cultures of each strain were grown to mid-log phase, then inoculated at a 1:100 ratio into 20 mL of MSgg medium, and incubated at 37°C with shaking at 200 rpm. Once the OD_{600} of the cells reached 1 and 2.5, cells were collected for RNA extraction. Extracted RNA from each strain was reverse-transcribed into cDNA for qPCR.

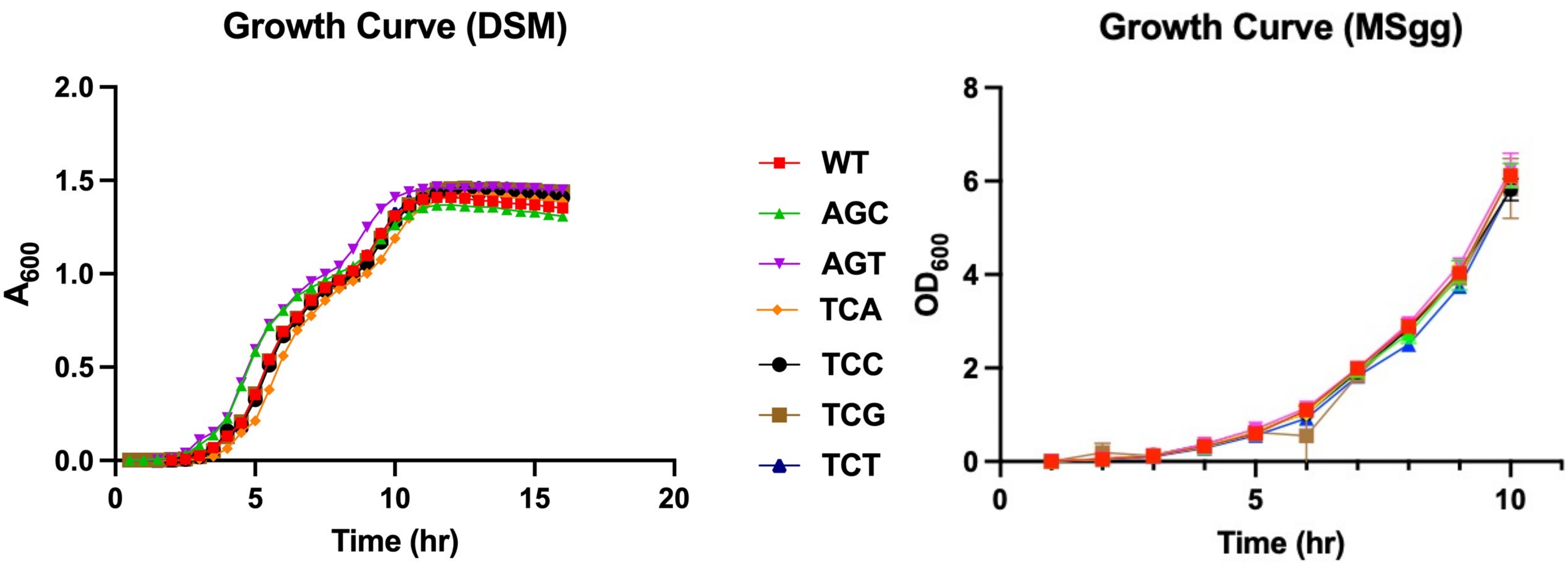
Growth Curve (DSM)
2.0
1.5
1.0
0.5
0.0
A600
0
5
10
15
20
Time (hr)
WT
AGC
AGT
TCA
TCC
TCG
TCT
Growth Curve (MSgg)
8
6
4
2
0
OD600
0
5
10
Time (hr)

Figure S1

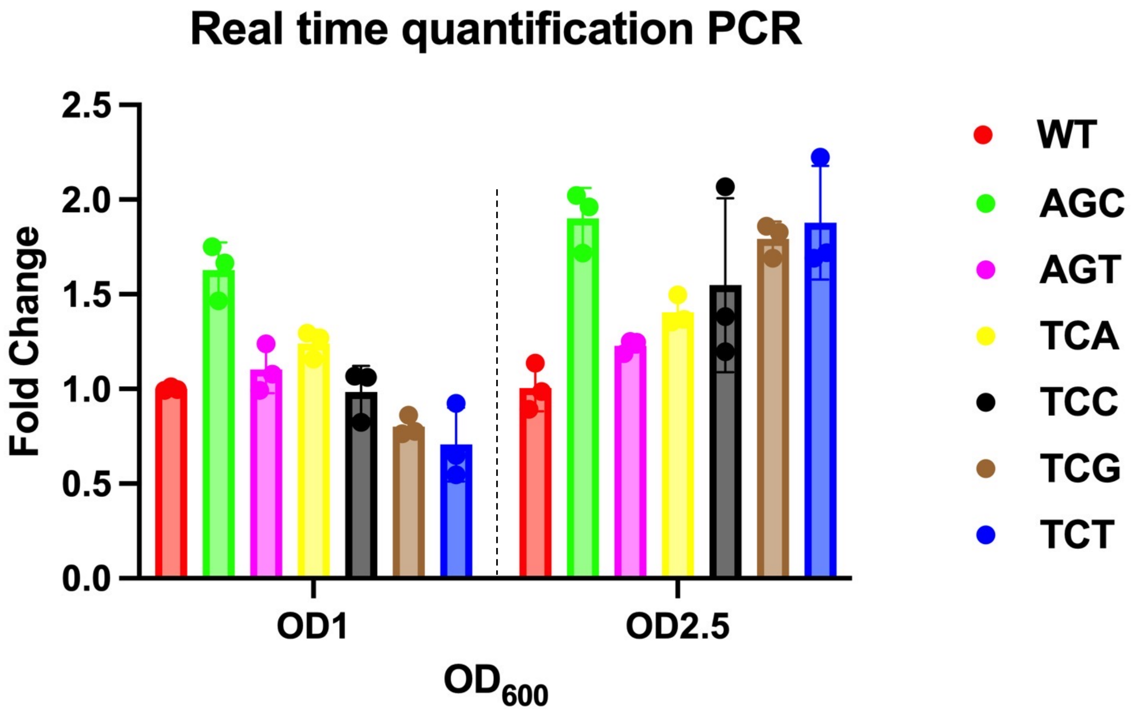
Real time quantification PCR
Fold Change
2.5
2.0
1.5
1.0
0.5
0.0
OD1
OD2.5
OD600
WT
AGC
AGT
TCA
TCC
TCG
TCT

Figure S2
